# Expression Of the Idh1 R132h Protein in Hepatopancreatobiliary Malignancies in A Single Center Population: Immunohistochemical Study

**DOI:** 10.22034/ijp.2026.2085869.3630

**Published:** 2026-08-01

**Authors:** Dina Sweed, Aya Hamdy Abdelbary, Yahya Fayed, Mohamed S.El Senbawy, Eman Sweed, Sara Mohamed Abd Elhamed

**Affiliations:** 1 Pathology Department, National Liver Institute, Menoufia University, Shebin Elkom, Menoufia, Egypt; 2 Hepatopancreaticobiliary Surgery, National Liver Institute, Menoufia University, Shebin Elkom, Menoufia, Egypt; 3 Clinical Oncology & Nuclear Medicine Department, Faculty of Medicine, Menoufia University, Shebin Elkom, Menoufia, Egypt; 4 Clinical Pharmacology Department, Faculty of Medicine, Menoufia University, Shebin Elkom, Menoufia, Egypt; 5 Clinical Pharmacology Department, Faculty of Medicine, Menoufia National University, Menoufia, Egypt

**Keywords:** Ampullary carcinoma, cholangiocarcinoma, gallbladder adenocarcinoma, hepatocellular carcinoma, IDH1, pancreatic ductal adenocarcinoma

## Abstract

**Background & Objective:**

Isocitrate dehydrogenase 1 (IDH1) mutations are promising therapeutic targets for various cancers. The study aims to elucidate the frequency of IDH1 expression in hepatocellular carcinoma (HCC), biliary tract cancer (BTC), and pancreatic ductal adenocarcinoma (PDAC), as well as its clinicopathological association in the Egyptian population.

**Methods:**

A retrospective cohort comprising 61 HCC, 65 BTC, and 29 PDAC cases was analyzed, with corresponding available control tissues included. IDH1 R132H protein expression was assessed using immunohistochemical analysis.

**Results:**

High IDH1 expression was observed in 16.4% of HCC, 30% of BTC, and 6.9% of PDAC, with a lower frequency in intrahepatic compared with extrahepatic cholangiocarcinoma (CC). Elevated IDH1 levels were observed in HCC arising in interferon-treated HCV patients, particularly in the absence of underlying cirrhosis (P = 0.03 and P = 0.002). High IDH1 expression was associated with lymphovascular invasion in iCC and with the absence of perineural invasion in PDAC (P = 0.05 and P = 0.009). IDH1 expression did not emerge as a significant independent predictor of overall survival in the HCC group.

**Conclusion:**

High IDH1 expression was observed in subsets of HCC and BTC, and to a lesser extent in PDAC, with no robust prognostic marker in these malignancies. The inconsistent findings regarding IDH1 expression in extrahepatic versus intrahepatic CC may reflect differences in ethnicity, disease etiology, environmental factors, sample size, and methodological approaches.

## Introduction

Liver cancer accounts for 7.8% of all cancer-related fatalities worldwide, making it the third most prevalent cause of cancer mortality(1). Biliary tract cancer (BTC) can develop anywhere along the biliary ductal system from the intrahepatic ducts to the extrahepatic biliary system, including the gallbladder and the ampulla of Vater(2). Pancreatic ductal adenocarcinoma (PDAC) continues to be one of the most deadly cancers(1).

Isocitrate dehydrogenase 1 (IDH1) is one of three IDH isozymes, alongside IDH2 and IDH3, encoded by the genes IDH1, IDH2, IDH3A, IDH3B, and IDH3G(3). Mutations in IDH genes are typically heterozygous and lead to a loss of normal enzymatic function, resulting in the abnormal accumulation of 2-hydroxyglutarate (2-HG). This metabolite induces widespread epigenetic alterations in histone and DNA methylation, thereby promoting tumorigenesis(4,5).

Mutations in the IDH1 gene are relatively rare across human cancers, occurring in about 1% to 2% of all cases according to large genomic datasets(6). Their distribution varies significantly by tumor type, with most IDH1 mutations found in central nervous system tumors (68.7%). Fewer mutations are seen in hematopoietic/lymphoid tissues (14.2%) and other tissues, each accounting for less than 10%. Current cancer research places significant emphasis on developing inhibitors against mutant IDH1. IDH1 inhibitors such as AG-120 (ivosidenib) have a potential role in the management of advanced or metastatic CCA and potentiate chemotherapy efficacy in pancreatic cancer(7,8). On the other hand, the IDH1 agonist scutellarin exerts antitumor effects in HCC progression(9).

Therefore, this study aimed to investigate the protein expression of IDH1 in hepatocellular carcinoma (HCC), BTC, including intrahepatic cholangiocarcinoma (iCC) and extrahepatic cholangiocarcinoma (gallbladder adenocarcinoma [GBC] and ampullary adenocarcinoma [AAC]), as well as PDAC in the Egyptian population, and to clarify its potential prognostic role in these cancers.

## Materials and Methods

This retrospective study comprised formalin-fixed, paraffin-embedded tissue samples from patients diagnosed with 61 HCC cases, 65 BTC cases (iCC, 24 cases; GBC, 13 cases; AAC, 28 cases), and 29 PDAC cases, all retrieved after surgical resection from the archives of the Pathology Department. Control samples were taken from the available non-tumorous tissues, which included 20 liver, 7 gallbladder, 12 duodenum, and 9 pancreatic specimens. All specimens were retrieved from the archives of the Pathology Department between 2024 and 2025, and the study commenced in June 2025.

This study adhered to the ethical standards outlined in the Declaration of Helsinki and was approved by the Research Ethics Committee of the National Liver Institute, Menoufia University (IRB No. NLI IRB 00014014/FWA00034015).

### Exclusion Criteria

Cases that had received neoadjuvant chemotherapy before surgery, those with mixed histologic subtypes, incomplete clinical data, or insufficient paraffin material were excluded (four HCC cases, one iCC case, two GBC cases, two AAC cases, and one PDAC case) to ensure sample uniformity and data reliability.

### Data Collection and Histopathological Evaluation

Clinical, serological, and radiological information was retrieved from patients’ medical records. Histopathological assessment of all tumor specimens was performed according to the 5th edition of the WHO Classification of Tumours of the Digestive System, and tumor staging was classified following the 8th edition of the American Joint Committee on Cancer (AJCC) criteria(10,11).

### Immunohistochemistry Technique

Tissue microarray (TMA) blocks were constructed for each tumor group. Sections of 4 µm thickness were cut, placed on positively charged slides, and processed using the streptavidin–biotin amplification method. The primary antibody was mouse monoclonal anti-IDH1 R132H (Code: QM002) and was sourced from Quartett, Am Mühlenberg, Germany. After deparaffinization and rehydration, antigen retrieval was achieved using a high-pH EDTA buffer, followed by cooling at room temperature. Slides were then incubated overnight at 4°C with the primary antibody. The secondary antibody was applied using Ultravision’s detection system (anti-polyvalent horseradish peroxidase/3,3′-diaminobenzidine [DAB]; Neomarker, USA). Color visualization was performed with DAB, and counterstaining was done using Mayer’s hematoxylin. Appropriate positive and negative controls were included for quality assurance.

### Immunohistochemistry Interpretation

Two histopathologists independently assessed the IDH1 H-score as the average of three different tumor areas using the TMA technique. The H-score method was used for semi-quantitative evaluation, calculated as follows: (1 × % weak staining) + (2 × % moderate staining) + (3 × % strong staining), as in a prior study(12). High IDH1 expression was defined as strong, diffuse cytoplasmic staining in more than 200 tumor cells, while low expression was classified as staining in ≤200 tumor cells(12). Diffuse immunopositivity observed in all tumor cells is regarded as indicative of an IDH1 mutation, reflecting its role as an early clonal event(13).

### Statistical Analysis

Statistical analysis was performed using SPSS software version 26. Descriptive statistics, including mean, standard deviation, median, and percentages, were used to summarize the data. Analytical tests such as the chi-square test, Monte Carlo test, and t test were applied as appropriate. A P value < 0.05 was considered statistically significant, while P ≤ 0.01 indicated high statistical significance.

Overall survival (OS) was assessed using the Kaplan–Meier method, and comparisons between groups were made using the log-rank test. To identify independent prognostic factors, Cox proportional hazards regression analysis was performed for multivariate evaluation, calculating hazard ratios (HRs) and 95% confidence intervals (CIs). Variables with P < 0.05 were deemed statistically significant.

## Results

### The Clinicopathological Data of the Studied Groups

The HCC cases were categorized into three subgroups according to prior HCV treatment history: 12 patients had no previous treatment (19.7%), 29 received direct-acting antiviral agents (DAAs) (47.5%), and 8 were treated with interferon (IFN) (13.1%).

Background liver tissue was cirrhotic in 47 cases (77.1%) and noncirrhotic in 14 cases (22.9%).

Recurrence data were available for 49 patients, among whom 18 (36.7%) showed tumor recurrence.

The clinicopathological features of the studied group are summarized in Supplementary Table 1.

### Immunohistochemical Expression of IDH1 in the Studied Control and Cancer Groups

**Fig. 1:**
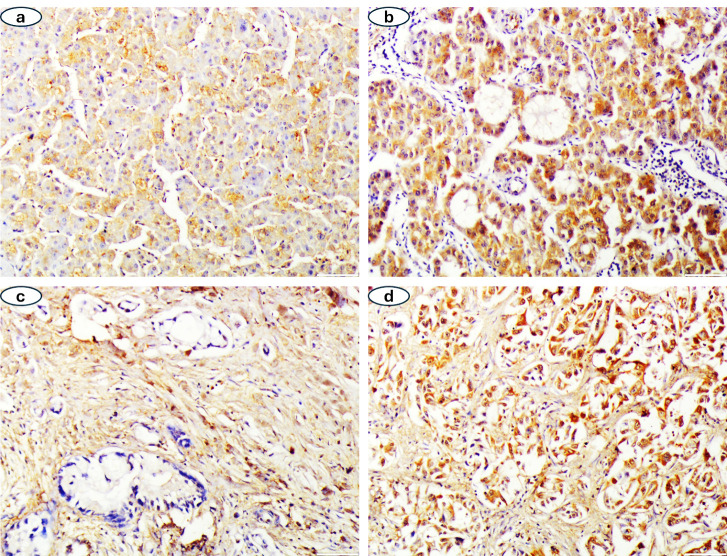
Immunohistochemical expression of IDH1 in the HCC and iCC groups. (A) Low cytoplasmic IDH1 expression in HCC. (B) High cytoplasmic IDH1 expression in HCC. (C) Low cytoplasmic IDH1 expression in iCC. (D) High cytoplasmic IDH1 expression in iCC. (IHC, original magnification ×200, all).

**Fig. 2:**
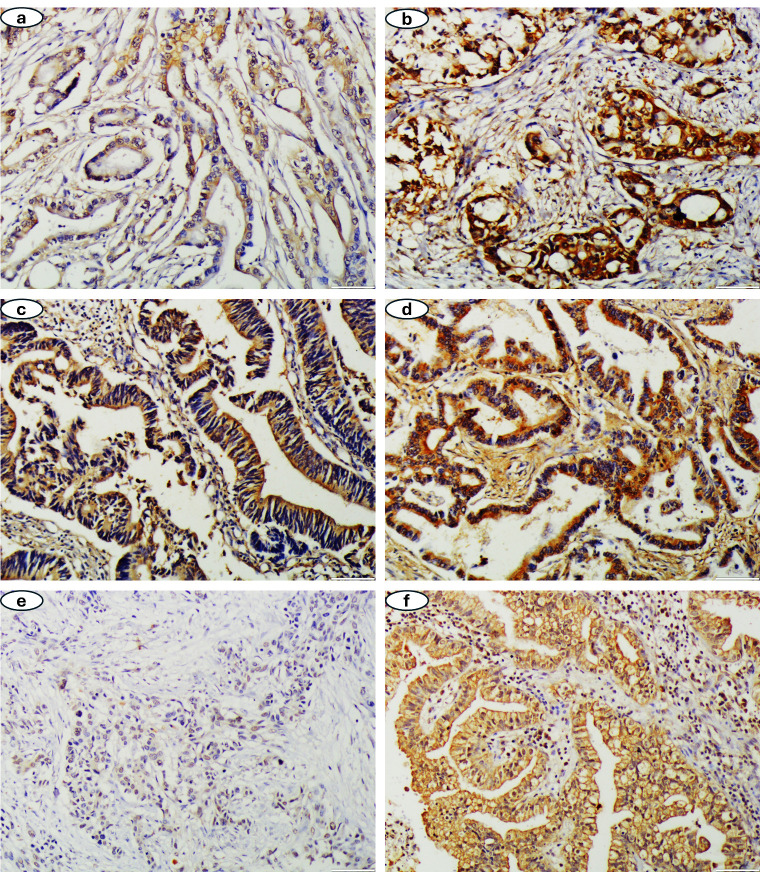
Immunohistochemical expression of IDH1 in the GBC, AAC, and PDAC groups. (A) Low cytoplasmic IDH1 expression in GBC. (B) High cytoplasmic IDH1 expression in GBC. (C) Low cytoplasmic IDH1 expression in AAC. (D) High cytoplasmic IDH1 expression in AAC. (E) Low IDH1 cytoplasmic expression in PDAC, d) High IDH1 cytoplasmic expression in PDAC (IHC, 200x, all).

All control group samples exhibited low IDH1 expression. High IDH1 expression was detected in 17.4% of all cancer cases (Figs. 1 and 2).

Low cytoplasmic IDH1 expression in PDAC. (F) High cytoplasmic IDH1 expression in PDAC. (IHC, original magnification ×200, all).

**Fig. 3:**
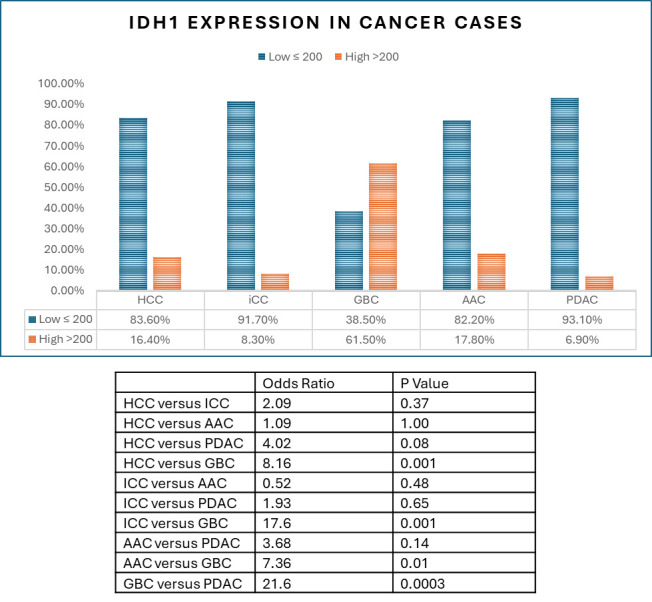
IDH1 expression in the studied cancer cases.

In addition, high IDH1 expression was observed in 16.4% of HCC, 30% of BTC, and 6.9% of PDAC. IDH1 expression in the GBC group was significantly higher than that in the other groups (Fig. 3).

### Relationship Between IDH1 Expression and Clinicopathological Parameters in the HCC Group

Elevated IDH1 expression was observed significantly more often in HCC arising in IFN-treated patients than in those treated with DAAs or in untreated HCV-associated HCC cases (P = 0.03).

Additionally, IDH1 expression was higher in background noncirrhotic liver tissue than in cirrhotic liver tissue from HCC cases (P = 0.02) (Table 1).

**Table 1:** The relationship between IDH1 expression and the clinicopathological data of the HCC group studied.

Clinical data	IDH1 in HCC		Test/P-value
Low (n=51)	High (n=10)
Age (mean ±SE)	56.80± 0.82	58.00± 1.57	T test: 0.55
Gender			
Male	47 (92.2%)	10 (100%)	Chi2: 0.36
Female	4 (7.8%)	0 (0%)	
AFP	944.85± 457.23	675.07± 52.04	T test: 0.81
Tumor focality			
Single	38 (74.5%)	9 (90%)	Chi2: 0.28
Multiple	13 (24.5%)	1 (10%)	
Tumor size ≥5			
Less	27 (52.9%)	6 (60%)	Chi2: 0.68
More	24 (47.1%)	4 (40%)	
Tumor stage			
Early	46 (90.2%)	8 (80%)	Chi2: 0.35
Late	5 (9.8%)	2 (20%)	
PNI			
Positive	1 (2%)	1 (10%)	Chi2: 0.19
Negative	50 (98%)	9 (90%)	
LVI			
Positive	24 (47.1%)	3 (30%)	Chi2: 0.32
Negative	27 (52.9%)	7 (70%)	
Previous HCV TTT#			
No	11 (27.5%)	1 (11.2%)	Chi2: 0.03*
DAAs	25 (62.5%)	4 (44.4%)	
IFN	4 (10%)	4 (44.4%)	
Adjacent non-tumor liver tissue			
Cirrhotic	42 (82.4%)	5 (50%)	Chi2: 0.02*
Non-cirrhotic	9 (17.6%)	5 (50%)	
Tumor grade			
Grade 1	3 (5.9%)	0 (0%)	Chi2: 0.46
Grade 2	31 (60.8%)	8 (80%)	
Grade 3	17 (33.3%)	2 (20%)	
Recurrence			
Free	25 (62.5%)	6 (66.7%)	Chi2: 0.81
Yes	15 (37.5%)	3 (33.3%)	

# Data were unavailable for 12 patients because no conclusive medical records documenting the initial HCV treatment received were available.

IDH1: Isocitrate dehydrogenase 1, HCC: Hepatocellular carcinoma, HCV: Hepatitis C virus, AFP: Alpha-fetoprotein, TTT: Treatment, DAAs: Direct-acting antiviral agents, IFN: Interferon, PNI: Perineural invasion, LVI: Lymphvascular invasion.

Relationship Between IDH1 Expression and Clinicopathological Parameters in the iCC Group

High IDH1 expression was associated with positive lymphovascular invasion in the iCC group (P = 0.05) (Table 2).

**Table 2:** The relationship between IDH1 expression and the clinicopathological data of the iCC group studied.

Clinical data	IDH1 in iCC		Test/P-value
Low (n=22)	High (n=2)
Age (Mean ±SD)	53.59± 2.44	53.50± 8.50	T test: 0.99
Gender			
Male	13 (59.1%)	2 (100%)	Chi2: 0.25
Female	9 (40.9%)	0 (0%)	
CA19-9	2742.50± 2471.72	38.00±0.00	U: 0.72
Tumor focality			
Single	18 (81.8%)	2 (100%)	Chi2: 0.50
Multiple	4 (18.2%)	0 (0%)	
Tumor size ≥5			
Less	10 (45.5%)	1 (50%)	Chi2: 0.90
More	12 (54.5%)	1 (50%)	
Tumor Stage			
Early	19 (86.4%)	2 (100%)	Chi2: 0.57
Late	3 (13.6%)	0 (0%)	
PNI			
Positive	8 (36.4%)	1 (50%)	Chi2: 0.70
Negative	14 (63.6%)	1 (50%)	
LVI			
Positive	7 (31.8%)	2 (100%)	Chi2: 0.05
Negative	15 (68.2%)	0 (0%)	
Tumor grade			
Grade 1	8 (36.4%)	1 (50%)	Chi2: 0.70
Grade 2	14 (63.6%)	1 (50%)	
Grade 3	0 (0%)	0 (0%)	

IDH1: Isocitrate dehydrogenase 1, iCC: Intrahepatic cholangiocarcinoma, CA19-9: Carbohydrate Antigen 19-9, PNI: Perineural invasion, LVI: Lymphvascular invasion.

Relationship Between IDH1 Expression and Clinicopathological Parameters in the GBC and AAC Groups

In the GBC and AAC groups, no significant relationship was found between IDH1 expression and any clinicopathological parameters (Supplementary Table 2).

Relationship Between IDH1 Expression and Clinicopathological Parameters in the PDAC Group

In the PDAC group, high IDH1 expression was significantly linked to the absence of perineural invasion (P = 0.009) (Table 3).

**Table 3:** Relationship between IDH1 expression and clinicopathological parameters in the PDAC group

Clinical data (PDAC)	IDH1 in PDAC		Test/P-value
Low (n = 27)	High (n = 2)
Age	58.96± 1.88	60.00± 5.00	T test 0.88
Gender			
Male	23 (85.2%)	2 (100%)	Chi2 0.55
Female	4 (14.8%)	0 (0%)	
Tumor focality			
Single	27 (100%)	2 (100%)	----
Multiple	0 (0%)	0 (0%)	
Tumor size ≥5			
Less	19 (70.4%)	2 (100%)	Chi2 0.36
More	8 (29.6%)	0 (0%)	
Tumor Stage			
Early	14 (51.9%)	2 (100%)	Chi2 0.18
Late	13 (48.1%)	0 (0%)	
PNI			
Positive	22 (81.5%)	0 (0%)	Chi2 0.009*
Negative	5 (18.5%)	2 (100%)	
LVI			
Positive	7 (25.9%)	0 (0%)	Chi2 0.40
Negative	20 (74.2%)	2 (100%)	
Tumor grade			
Grade 1	7 (25.9%)	0 (0%)	------------
Grade 2	16 (59.3%)	2 (100%)	
Grade 3	4 (14.8%)	0 (0%)	

IDH1: Isocitrate dehydrogenase 1, PDAC: Pancreatic ductal adenocarcinoma, PNI: Perineural invasion, LVI: Lymphvascular invasion.

### Overall Survival

In the multivariate analysis, IDH1 expression did not emerge as a significant independent predictor of overall survival in the HCC group. Conversely, larger tumor size was consistently associated with poorer prognosis and represented a significant adverse prognostic factor in both the HCC and combined PDAC/AAC cohorts (log-rank P = 0.019 and P < 0.001, respectively) (Table 4).

**Table 4:** Univariate Kaplan-Meier/log-rank survival analysis of clinicopathological parameters in HCC and AAC/PDAC groups.

HCC group				
Clinical data	Mean survival time (95% CI)	SE	Log rank	P-value
Tumor focality				
Single	44.35	2.55	0.004	0.95
Multiple	47.90	3.16		
Tumor size ≥5				
Less	48.24	2.73	5.53	0.019*
More	40.50	2.88		
Tumor Stage				
Early	45.18	2.24	0.06	0.80
Late	46.00	6.16		
LVI				
Positive	43.79	3.24	0.04	0.83
Negative	46.83	2.69		
Previous HCV TTT				
No	43.73	3.30	1.18	0.55
DAA	46.42	2.88		
IFN	43.91	6.25		
Adjacent non-tumor liver tissue				
Non-cirrhotic	45.10	3.71	0.241	0.62
Cirrhotic	45.21	2.46		
Tumor grade				
Grade 1	43.00	5.00	0.70	0.70
Grade 2	46.41	2.79		
Grade 3	44.22	3.65		
Recurrence				
Free	44.30	2.84	0.02	0.87
Yes	46.70	3.07		
IDH1 expression				
Low	44.61	2.11	1.298	0.25
High	52.50	9.03		
PDAC and AAC group				
Clinical data	Mean survival time (95% CI)	SE	Log rank	P-value
Tumor size ≥5				
Less	30.87	2.65	11.00	<0.001*
More	15.00	0.00		
Tumor Stage				
Early	30.35	2.83	0.31	0.57
Late	21.75	1.94		
PNI				
Positive	29.25	5.25	0.01	0.91
Negative	28.28	4.05		
LVI				
Positive	30.18	3.03	0.506	0.47
Negative	22.66	1.08		

IDH1: Isocitrate dehydrogenase 1, HCC: Hepatocellular carcinoma, AAC: Ampullary adenocarcinoma, PDAC: Pancreatic ductal adenocarcinoma, HCV: Hepatitis C virus, TTT: Treatment, DAAs: Direct-acting antiviral agents, IFN: Interferon, PNI: Perineural invasion, LVI: Lymphvascular invasion.

## Discussion

Our findings demonstrate that high IDH1 protein expression is present in a subset of HCC and BTC, with a lower frequency in iCC compared with eCC, and is rare in pancreatic cancer. Although IDH1 protein expression did not independently affect survival, it was associated with adverse prognostic features in iCC and with more favorable prognostic characteristics in PDAC. In HCC, IDH1 expression appeared to be influenced by prior HCV treatment and the underlying liver background.

In a cohort of Chinese solid tumors, IDH1/IDH2 mutations were found most frequently in BTC and liver cancers(14). While some cohorts report higher IDH1 mutation rates in iCC and lower rates in other biliary and liver cancers, the absolute differences in overexpression rates between AAC, HCC, iCC, and PDAC are generally small and often not statistically significant(15,16). In addition, large-scale studies indicate that, despite observed trends in IDH1 expression frequencies among different tumor types, these differences do not reach statistical significance, likely due to overlapping ranges and sample size limitations(17,16).

Lee et al. reported that IDH1 mutations occur more frequently in HCC than in iCC(18). In contrast, previous research indicates that IDH1 mutations and overexpression occur most frequently in iCC, but they can also appear in HCC and, to a lesser extent, in GBC and eCC(19). Data from The Cancer Genome Atlas indicate that the overall IDH1 mutation frequency in HCC is only 1.6%. Moreover, the mutation allele frequency of IDH1 in HCC is typically very low, making it difficult to detect using conventional methods, and it may be associated with later stages of tumor progression(18).

The current study found that IFN-treated HCV cases associated with HCC display significantly higher IDH1 expression compared with those treated with DAAs or untreated HCV-related HCC. Mutations in the IDH1 gene can suppress the IFN antiviral response, while wild-type IDH1 (wtIDH1) and targeting the mutant form can lead to increased IFN expression. Gene expression data indicate that mutant IDH1 (mIDH1) inhibition promotes features of hepatocyte differentiation in tumor cells while also provoking rapid changes in tumor-immune interplay and IFN pathway activation(20). Conversely, some studies show that inhibiting mIDH1 can stimulate IFN-γ production by recruiting immune cells(21). Moreover, IDH1 activity in liver diseases can be modulated by various posttranslational modifications, including acetylation, phosphorylation, and crotonylation(22). Therefore, IDH1 expression could be a potential marker for specific HCC subgroups, suggesting a link to distinct molecular pathways in hepatocarcinogenesis.

The current study found that high IDH1 expression occurred in eCC cases compared with iCC cases. Our results are discordant with previous studies. Murugan et al. demonstrated that IDH1 mutations are frequent in iCC but rare in eCC and AAC(23). Evaluation of an expanded GBC and BTC cohort demonstrated that IDH1/IDH2 mutations are restricted to iCC and were not detected in eCCA or GBC(15). The inconsistencies may reflect underlying experimental bias, heterogeneity in geographic study location, the various clinical stages examined, and insufficient sample sizes(19).

The present study found high IDH1 expression in only two PDAC cases, consistent with previous reports showing that IDH1 mutations are extremely uncommon in pancreatic cancer(24). This rarity may reflect the inability of PDAC tumors, characterized by a particularly harsh microenvironment, to accommodate IDH1 mutations(25). Under the nutrient-deprived conditions typical of these tumors, PDAC cells depend on wtIDH1-driven isocitrate oxidation to produce NADPH and α-KG, supporting antioxidant defenses and mitochondrial function(26). Surprisingly, inhibitors originally developed for mutant IDH1, such as ivosidenib, can also suppress wtIDH1 under PDAC-like conditions(8). Independent studies in prostate cancer have validated many of these observations(27). In addition, wtIDH1 knockout leads to a decrease in the aggressive features of colon cancer cells and could be used as a therapeutic target(28).

Regarding its prognostic role, elevated IDH1 expression was associated with lymphovascular invasion in iCC and with the absence of perineural invasion in PDAC, while no significant associations were observed in HCC or eCC. Previous studies in iCC have reported inconsistent associations between mIDH1 status and demographic or prognostic pathological variables(19). A previous study found that the mutation frequency of IDH1 in HCC may be associated with advanced stages of tumor progression(18). IDH1 mutations are generally associated with an adverse prognosis in hematological malignancies and chondrosarcoma of bone and a favorable prognosis in glioblastoma(29–31). The prognostic impact of IDH1 mutations may be influenced by distinct tumor microenvironmental factors or variations in mutant IDH-driven DNA hypermethylation and context-dependent activities of IDH1 across tumor types(31).

## Conclusions

Current research indicates that high IDH1 expression is observed in subsets of HCC and BTC, and to a lesser extent in PDAC. The inconsistent findings regarding IDH1 expression in extrahepatic versus intrahepatic CC may reflect differences in ethnicity, disease etiology, environmental factors, sample size, and methodological approaches. IDH1 expression alone does not appear to be a robust prognostic marker in hepatobiliary malignancies.

## Data Availability

The data that support the findings of this study are available from the corresponding author upon request.

## Supplementary Material

Supplementary Tables 1–2 Clinicopathological features of the cancer groups, and the relationship between IDH1 expression and clinicopathological data in GBC and AAC.
