# Supplementary material for: Expression Of the Idh1 R132h Protein in Hepatopancreatobiliary Malignancies in A Single Center Population: Immunohistochemical Study
Source: Iran J Pathol. 2026 Aug 1;21(4):531–42. doi: 10.22034/ijp.2026.2085869.3630 (PMC13477784; doi:10.22034/ijp.2026.2085869.3630)
Supplement: Supplementary file 1 [file ijp-21-4-531-s001.pdf]

Iranian Journal of Pathology | ISSN: 2345-3656

Expression Of the Idh1 R132h Protein in Hepatopancreatobiliary Malignancies in A Single Center Population: Immunohistochemical Study

Supplementary Table 1. The clinicopathological features of the cancer groups studied.

Clinicopathological data	HCC (n=61)	iCC (n=24)	GBC (n=13)	PDAC (n=29)	AAC (n=28)
**Age (mean±SEM)**	57.00±0.73	53.58±2.29	55.54±2.26	59.03±1.77	57.86±1.75
**Gender**					
**Male**	57 (93.4%)	15 (62.5%)	6 (46.2%)	25 (86.2%)	18 (64.3%)
**Female**	4 (6.7%)	9 (37.5%)	7 (53.8%)	4 (13.8%)	10 (35.7%)
**Virology**					
**HCV**	61 (100%)	8 (33.3%)		2 (28.6%)	5 (33.3%)
**Non-HCV**	0 (0%)	16 (66.7%)		5 (71.4%)	10 (66.7%)
**Tumor markers (median±IQR)**					
**AFP**	582.97±703.26	4.00±9.82			
**CA19-9**	81.85±79.65	100±798		55.90±191.00	74.00±328.00
**Tumor focality**					
**Single**	47 (77%)	20 (83.3%)	12 (92.3%)	29 (100%)	28 (100%)
**Multiple**	14 (23%)	4 (16.7%)	1 (7.7%)	0 (0%)	0 (0%)
**Tumor size ≥5**					
**Less**	33 (54.1%)	11 (45.9%)	5 (38.5%)	21 (72.4%)	25 (89.3%)
**More**	28 (45.9%)	13 (54.2%)	8 (61.5%)	8 (27.6%)	3 (10.7%)
**Tumor Stage**					
**Early**	54 (88.5%)	21 (87.5%)	9 (69.2%)	16 (55.2%)	19 (67.9%)
**Late**	7 (11.5%)	3 (12.5%)	4 (30.8%)	13 (44.8%)	9 (32.1%)
**PNI**					
**Positive**	2 (3.3%)	15 (62.5%)	6 (45.2%)	7 (24.1%)	5 (17.9%)
**Negative**	59 (96.7%)	9 (37.5%)	7 (53.8%)	22 (75.9%)	23 (82.1%)
**LVI**					
**Positive**	27 (44.3%)	9 (37.5%)	5 (38.5%)	7 (24.1%)	10 (35.7%)
**Negative**	34 (55.7%)	15 (62.5%)	8 (61.5%)	22 (75.9%)	18 (64.3%)

HCC: Hepatocellular carcinoma, iCC: Intrahepatic cholangiocarcinoma, GBC: Gallbladder carcinoma, AAC: Ampullary adenocarcinoma, PDAC: Pancreatic ductal adenocarcinoma, HCV: Hepatitis C virus, AFP: Alpha-fetoprotein, CA19-9: Carbohydrate Antigen 19-9, PNI: Perineural invasion, LVI: Lymphvascular invasion.

Supplementary Table 2: The relationship between IDH1 expression and the clinicopathological data of the GBC and AAC groups studied.

Clinical data	IDH1 in GBC		Test/P-value	IDH1 in AAC		Test/P-value
	Low (n = 5)	High (n = 8)		Low (n = 23)	High (n = 5)	
**Age**	57.60±7.50	54.25±8.79	T test: 0.49	57.52± 1.82	59.40± 5.58	T test: 0.69
**Gender**						
**Male**	1 (20%)	5 (62.5%)	Chi$^{2:}$ 0.13	14 (60.9%)	4 (80%)	Chi$^{2:}$ 0.41
**Female**	4 (80%)	3 (37.5%)		9 (39.1%)	1 (20%)	
**Tumor focality**						
**Single**	4 (80%)	8 (100%)	Chi$^{2:}$ 0.18	23 (100%)	5 (100%)	Chi$^{2:}$ 1
**Multiple**	1 (20%)	0 (0%)		0 (0%)	0 (0%)	
**Tumor size ≥5**						
**Less**	1 (20%)	4 (50%)	Chi$^{2:}$ 0.27	21 (91.3%)	4 (80%)	Chi$^{2:}$ 0.45
**More**	4 (80%)	4 (50%)		2 (8.7%)	1 (20%)	
**Tumor Stage**						
**Early**	4 (80%)	5 (62.5%)	Chi$^{2:}$ 0.50	17 (73.9%)	2 (40%)	T test: 0.47
**Late**	1 (20%)	3 (37.5%)		6 (26.1%)	3 (60%)	
**PNI**						
**Positive**	3 (60%)	4 (50%)	Chi$^{2:}$ 0.72	19 (82.6%)	4 (80%)	Chi$^{2:}$ 0.14
**Negative**	2 (40%)	4 (50%)		4 (17.4%)	1 (20%)	
**LVI**						
**Positive**	2 (40%)	3 (37.5%)	Chi$^{2:}$ 0.92	9 (39.1%)	1 (20%)	Chi$^{2:}$ 0.41
**Negative**	3 (60%)	5 (62.5%)		14 (60.9%)	4 (80%)	
**Tumor grade**						
**Grade 1**	0 (0%)	3 (37.5%)	Chi$^{2:}$ 0.23	2 (8.7%)	1 (20%)	Chi$^{2:}$ 0.69
**Grade 2**	3 (60%)	4 (50%)		20 (87%)	4 (80%)	
**Grade 3**	2 (40%)	1 (12.5%)		1 (4.3%)	0 (0%)	

IDH1: Isocitrate dehydrogenase 1, GBC: Gallbladder carcinoma, AAC: Ampullary adenocarcinoma, PNI: Perineural invasion, LVI: Lymphvascular invasion.
